# Welding-Induced Heterogeneity Promotes Gradient Nanostructuring in Laser-Welded 304 Stainless Steel Joints

**DOI:** 10.3390/nano16140859

**Published:** 2026-07-13

**Authors:** Tianzhang Zhao, Junping Zhu, Hongchuan Deng, Chuanchen Wang, Renwei Zhang, Qian Li, Yingwei Qi, Yantao Sun

**Affiliations:** 1 College of Aerospace Engineering, Shenyang Aerospace University, Shenyang 110136, China; zhaotianzhang@sau.edu.com (T.Z.); zxchw426@163.com (J.Z.); 13674119454@163.com (C.W.); ywqi1205sau@163.com (Y.Q.); 2 Institute of Materials Plainification, Liaoning Academic of Materials, Shenyang 110167, China; rwzhang@lam.ln.cn; 3 Chongqing Institute of Nanometals, Chongqing 401135, China; dhcfly@foxmail.com; 4 Department of Materials Science and Engineering, City University of Hong Kong, Hong Kong 999077, China; qli384@cityu.edu.hk

**Keywords:** laser welding, 304 stainless steel, surface mechanical rolling treatment, gradient nanostructure, martensitic transformation, chemical heterogeneity

## Abstract

Laser-welded stainless steel joints usually suffer from strain localization and premature failure in the weld metal (WM) due to microstructural heterogeneity introduced during welding. In this work, surface mechanical rolling treatment (SMRT) was applied to laser-welded 304 stainless steel plates to enhance the mechanical performance of the welded joints. Laser welding introduced multiple heterogeneous features in the WM, including local Ni compositional fluctuations, nanoscale oxide particles and heterogeneous grain structures. Among them, the local fluctuation of Ni concentration is considered to play a dominant role by locally modifying the stability of γ-austenite and promoting strain-induced martensitic transformation during SMRT. As a result, the WM exhibited more severe grain refinement and a stronger gradient nanostructure than base metal (BM) under identical processing conditions. The near-surface hardness of the WM reached ~500 Hv, which was noticeably higher than that of the BM. Uniaxial tensile tests revealed that the yield strength increased from ~350 MPa to ~700 MPa, while the ultimate tensile strength reached ~1000 MPa with ~40% elongation. More importantly, the fracture location shifted from the WM to the BM after SMRT. The enhanced martensitic transformation and gradient nanostructure effectively suppressed strain localization and improved the mechanical reliability of the welded joint.

## 1. Introduction

Austenitic stainless steels, particularly AISI 304 and 316L, have been extensively employed as important structural materials in various industries because of their excellent corrosion resistance, superior formability, and remarkable work-hardening capability [1,2,3,4]. With the increasing demand for lightweight and high-performance structures, laser welding has been widely used for joining stainless steels [5,6,7,8]. However, the rapid thermal cycle and localized melting/solidification during laser welding inevitably introduce strong microstructural heterogeneity in the weld metal (WM), including coarse columnar grains, residual stress, elemental segregation, and local phase transformation [9,10,11]. These heterogeneities often deteriorate the mechanical reliability of welded joints and promote strain localization, premature necking, and crack initiation during deformation.

Strengthening metallic materials through microstructural regulation has long attracted significant attention [12,13,14,15,16]. To improve the mechanical properties and service reliability of welded joints, various surface strengthening techniques, such as shot peening, laser shock peening, ultrasonic surface rolling, and ssurface mechanical rolling treatment (SMRT), have been extensively developed [17,18,19,20]. Among these methods, SMRT has attracted significant attention because it can produce gradient nanostructured layers with relatively large thickness while maintaining excellent surface quality [21,22,23]. Previous studies have demonstrated that gradient structures can effectively suppress strain localization and improve strain hardening capability by enabling progressive plastic deformation from the hard surface layer to the ductile interior [21,24]. Moreover, the high-density interfaces, nanoscale substructures, and residual compressive stresses generated during severe surface plastic deformation can significantly improve the fatigue resistance and mechanical stability of metallic materials [25,26].

For metastable austenitic stainless steels, strain-induced martensitic transformation plays a critical role during severe plastic deformation and mechanical loading [27,28,29]. In particular, the stability of γ-austenite strongly depends on the chemical composition, especially the Ni concentration. The progressive martensitic transformation not only contributes to strain hardening but also accelerates grain subdivision and nanostructure formation under severe plastic deformation [30,31,32]. Consequently, the coupling effect between deformation-induced martensitic transformation and gradient nanostructuring has become an important strategy for achieving superior mechanical properties in metastable stainless steels [23,33]. Although laser welding can introduce local elemental redistribution and compositional fluctuations due to rapid solidification, most previous studies mainly regard the WM as a mechanically weak region requiring mitigation [17,18,19]. Laser welding inevitably introduces multiple heterogeneous features into the weld metal, including heterogeneous grain structures resulting from directional solidification, local compositional fluctuations caused by rapid solidification, and nanoscale oxide particles. Although these heterogeneous features are generally regarded as detrimental to mechanical performance, their potential beneficial role in regulating subsequent gradient nanostructure formation has rarely been explored [8,34].

In this work, SMRT was applied to laser-welded 304 stainless steel joints to enhance their mechanical performance through gradient surface nanostructuring. The results reveal that laser welding induces multiple heterogeneous features into the weld metal, including heterogeneous grain structures, local compositional fluctuations, and nanoscale oxide particles. Among these heterogeneous features, local Ni compositional fluctuation is expected to play an important role and promote strain-induced martensitic transformation during SMRT. Consequently, the WM develops a finer gradient nanostructure and a higher hardness than the base metal (BM) under identical processing conditions. More importantly, the SMRT-treated welded joint exhibits excellent strength-ductility synergy, and the fracture no longer occurs in the WM after tensile deformation. This work provides a welding-assisted gradient strengthening strategy for improving the mechanical reliability of laser-welded stainless steel joints.

## 2. Experimental

**Figure 1:**
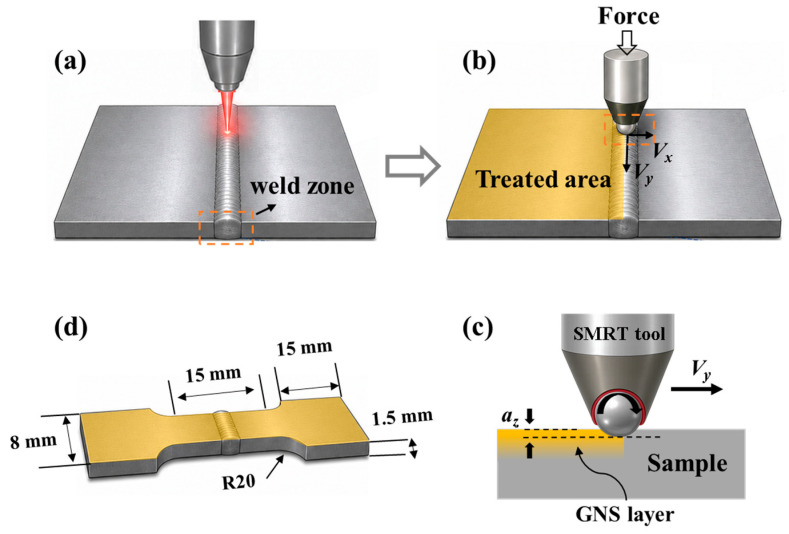
Schematic illustrations of (**a**) laser welding of 304 stainless steel plates, (**b**) SMRT, (**c**) the plastic deformation in the sample surface layer induced by rolling of the WC/Co cermet ball, and (**d**) the tensile specimen extraction and dimensions for mechanical testing.

A laser welding technique was employed to join 304 stainless steel plates with a thickness of ~1.5 mm, producing a weld seam width of ~1.5 mm (Figure 1a). The laser welding system utilized a 6 kW fiber laser (YLS-6000, IPG Photonics Corp., Oxford, MA, USA) operating at a wavelength of 1070 nm with a core diameter of 150 µm. Prior to welding, the 304 stainless steel plate was subjected to sandblasting to remove the surface oxide layer. The welding parameters were set as follows: a welding power of 3000 W, a welding speed of 20 mm/s, a linear scanning path, and argon as the shielding gas. Both surfaces of the welded stainless steel plates were ground and polished, followed by the SMRT (Figure 1b,c). The SMRT process was conducted using a method similar to that of our previous study [23,34]. A WC/Co cermet ball with a diameter of 8 mm was pressed into the sample surface and reciprocally rolled across the plate surface. The rolling speed was 4000 mm/min, and the interval between adjacent rolling paths was 30 μm. Both sides of the sample were treated with one rolling pass under a constant load of 80 kg.

In this work, a Qness Q10 A+ microhardness tester (QATM GmbH, Golling, Austria) was employed to measure the cross-sectional hardness distributions in both the WM and the BM of the 304 stainless steel plates. The indentation load was set at 50 g, with a constant dwell time of 10 s for all measurements. Dog-bone-shaped tensile specimens with a gauge length of 15 mm were extracted from the SMRT-treated plates by electrical discharge wire cutting, as shown in Figure 1d. The tensile loading direction was perpendicular to the weld direction. Uniaxial tensile tests were conducted at room temperature using a Z5.0TH universal testing machine (Zwick/Roell, Ulm, Germany) equipped at an initial strain rate of 5 × 10^−4^ s^−1^. A laser extensometer (Zwick/Roell, Ulm, Germany) was employed to monitor the strain during tensile loading.

The microstructural evolution was characterized using a scanning electron microscope (SEM, Apreo 2S, Thermo Fisher Scientific, Hillsboro, OR, USA) equipped with an electron backscatter diffraction (EBSD, Oxford Instruments, Abingdon, UK) detector. EBSD data were acquired via the Aztec Crystal acquisition software (Symmetry S3) under an accelerating voltage of 20 kV with a step size of 80 nm and 500 nm. And the elemental distribution across the WM was identified by electron probe microanalysis (EPMA, EPMA-1720, Shimadzu Corporation, Kyoto, Japan). EBSD and EPMA specimens were carefully ground and polished mechanically, followed by electropolishing for 45 s in an electrolyte consisting of 90 vol.% C_2_H_5_OH and 10 vol.% HClO_4_ at −20 °C. The nanoscale microstructure was characterized in detail using an FEI Talos F200X transmission electron microscope (TEM, Thermo Fisher Scientific, Hillsboro, OR, USA) operated at an accelerating voltage of 200 kV. TEM foils were first thinned to approximately 50 μm by mechanical grinding, followed by jet electropolishing using a Struers TenuPol-5 electropolishing (Struers A/S, Ballerup, Denmark) unit at 20 V and −20 °C.

## 3. Results and Discussion

### 3.1. Initial Microstructure and Chemical Heterogeneity of the Welded Joint

**Figure 2:**
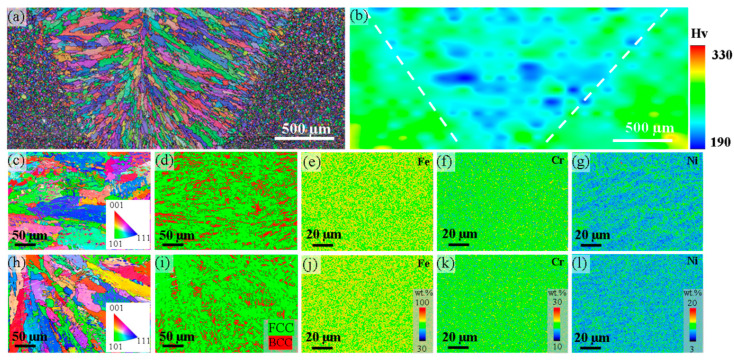
(**a**) EBSD inverse pole figure (IPF) map and (**b**) corresponding cross-sectional hardness contour map of the WM. (**c**,**d**) High-resolution IPF map and corresponding phase distribution map of the near-surface region in the WM (**e**–**g**) EPMA elemental distribution maps of Fe, Cr, and Ni in the near-surface region of the WM, respectively. (**h**,**i**) High-resolution IPF map and corresponding phase distribution map of the bottom region of the WM. (**j**–**l**) Corresponding EPMA elemental distribution maps of Fe, Cr, and Ni in the bottom region of the WM, respectively. The white dashed lines in (**b**) indicate the approximate location of the fusion boundary.

Figure 2a shows the EBSD inverse pole figure (IPF) map of the WM, which is characterized by coarse columnar grains resulting from the high thermal input and directional solidification during laser welding. The corresponding hardness distribution map is shown in Figure 2b. It can be observed that the WM contains several locally softened regions with slightly reduced hardness compared to the surrounding material (from ~250 Hv to ~190 Hv), indicating microstructural heterogeneity introduced during the welding process. The high-resolution EBSD maps shown in Figure 2c,d,h,i were acquired from representative regions near the top and bottom of the WM, respectively. Coarse austenitic grains contain numerous fine body-centered cubic (BCC) grains, with the BCC phase fraction reaching ~22.6%. The corresponding elemental distributions of Fe, Cr, and Ni in this region are presented in Figure 2e–g. The Fe and Cr elements exhibit relatively uniform distributions, whereas the Ni concentration shows pronounced compositional fluctuations. Figure 2h,i show the high-resolution EBSD microstructure near the bottom region of the WM. Similar to the near-surface region, the microstructure still consists of coarse austenitic grains containing fine BCC grains. However, the BCC phase fraction decreases from approximately 22.6% near the surface to approximately 12.4% in the bottom region. Correspondingly, the Ni compositional fluctuation also becomes less pronounced compared to that in the near-surface region shown in Figure 2g.

**Figure 3:**
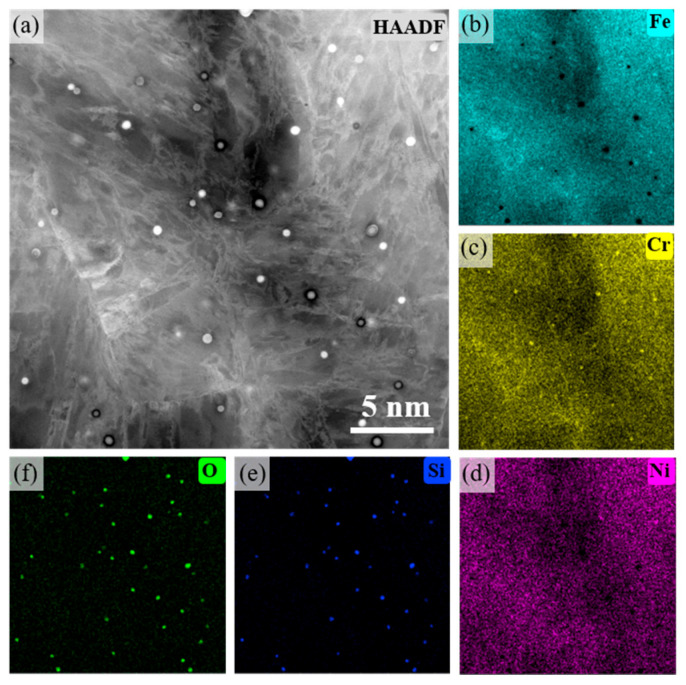
(**a**) HAADF-STEM image of the WM. (**b**–**f**) Corresponding EDS elemental maps of Fe, Cr, Ni, Si, and O, respectively, revealing the nanoscale chemical heterogeneity in the WM.

To further characterize the chemical heterogeneity introduced by laser welding, high-angle annular dark-field scanning transmission electron microscopy (HAADF-STEM) combined with EDS elemental mapping was performed in the WM, as shown in Figure 3. In addition to the microscale Ni compositional fluctuations revealed by EPMA (Figure 2g,l), nanoscale Cr–Si–O oxide particles were also observed. These oxide particles exhibit significant enrichment of Cr, Si and O, accompanied by local depletion of Fe and Ni. Among these heterogeneities, the local fluctuation of Ni concentration might play the dominant role in regulating the stability of γ-austenite because Ni is the principal austenite stabilizer in metastable 304 stainless steel.

### 3.2. Gradient Structure Formation After SMRT

**Figure 4:**
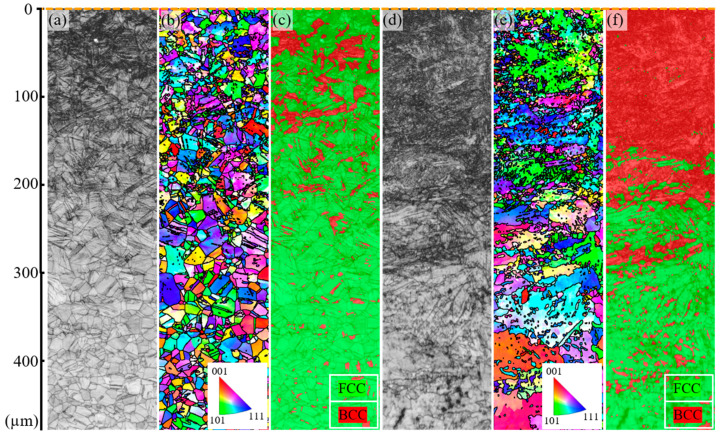
Typical cross-sectional band contrast images, inverse pole figure (IPF) maps, and phase distribution maps of the top surface layer (0–480 μm in depth) after SMRT: (**a**–**c**) BM and (**d**–**f**) WM.

Figure 4 compares the microstructural evolution in the BM and WM after SMRT under identical processing conditions. At a depth of approximately 400 μm from the treated surface, the BC map reveals a pronounced increase in deformation-induced defects (Figure 4a,b). Based on our previous TEM study of 304 stainless steel processed under SMRT, this region is expected to contain a high density of dislocations and nanoscale ε-martensite lamellae, which represent the initial stage of gradient nanostructure formation [23]. The ε-martensite plates progressively intersect and subdivide the original austenitic grains, which subsequently promotes the formation of α′-martensite and leads to significant grain refinement near the top surface (Figure 4c). The WM exhibits a similar gradient refinement process during SMRT. The coarse austenitic grains first undergo subdivision through the formation and interaction of ε-martensite plates (Figure 4d,e). With increasing plastic deformation toward the treated surface, the lamellar structures continuously refine and progressively transform into α′-martensite. However, compared with the BM, the WM shows a substantially stronger martensitic transformation. Within approximately 200 μm from the treated surface, the WM almost completely transforms into α′-martensite, as shown in Figure 4f. Correspondingly, the microstructure in the WM undergoes much more severe refinement.

It should be noted that the fundamental grain refinement mechanism in both the WM and BM remains essentially unchanged after SMRT. During deformation, ε-martensite preferentially forms through the glide of Shockley partial dislocations on {111}γ planes, and the intersecting ε-martensite laths gradually subdivide the original coarse austenite grains into refined lamellar domains [23,35,36]. With increasing strain and strain rate toward the treated surface, α′-martensite continuously nucleates from both ε-martensite and the γ-austenite matrix, following the typical γ→ε→α′ deformation-induced martensitic transformation. The combined effects of martensitic transformation, dislocation accumulation, and lamellar subdivision eventually lead to the formation of a heavily refined nanostructure near the top surface. More importantly, the welding process significantly enhances this refinement. As discussed above, laser welding introduces obvious Ni compositional fluctuations in the WM, might resulting in reduced γ-austenite stability. Since Ni is a strong austenite stabilizing element in 304 stainless steel, the local Ni-depleted regions are more susceptible to deformation-induced martensitic transformation during SMRT. The accelerated martensitic transformation further promotes lamellar subdivision and dynamic grain refinement, finally producing a thicker and harder gradient nanostructured layer in the WM. This result suggests that the welding-induced chemical heterogeneity could effectively assist the gradient nanostructuring process during severe surface plastic deformation.

### 3.3. Mechanical Properties and Fracture Behavior

**Figure 5:**
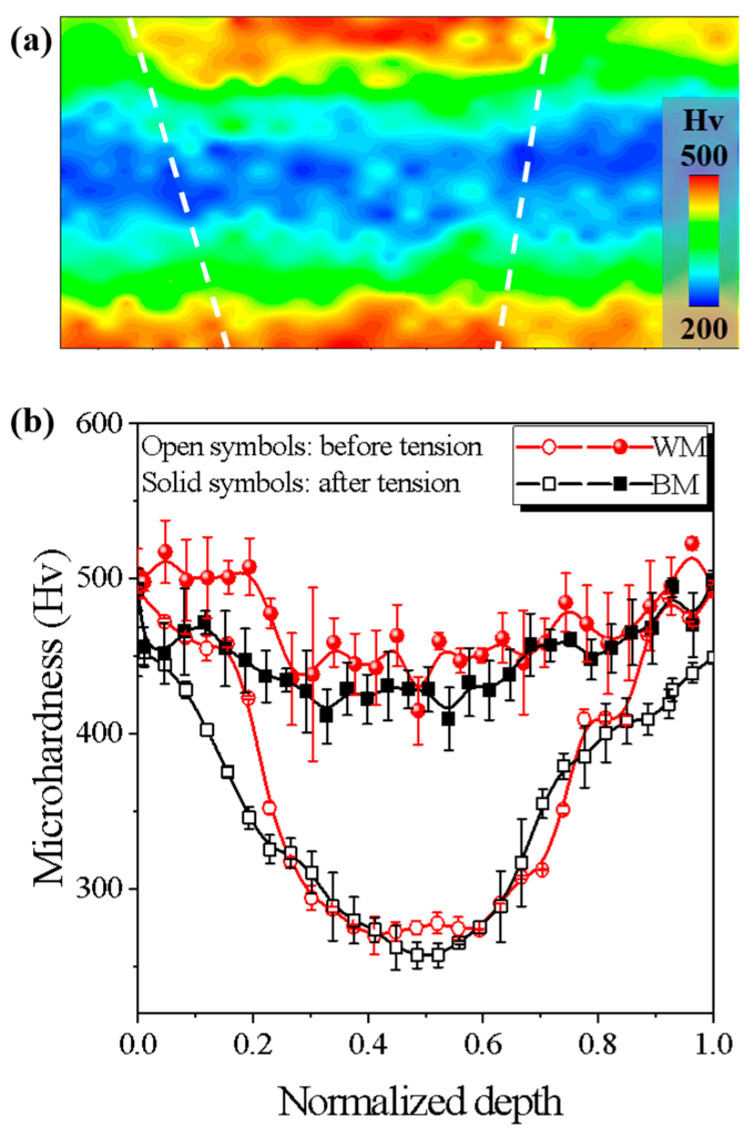
(**a**) Cross-sectional hardness contour map of the SMRT-treated welded joint. (**b**) Hardness profiles of the WM and BM before and after tensile deformation. The white dashed lines in (**b**) indicate the approximate location of the fusion boundary.

Figure 5a presents the hardness distribution map of the SMRT-treated welded joint. A typical sandwich-like hardness distribution forms along the depth direction, where the surface layers exhibit a high hardness of ~500 Hv, while the center region maintains a relatively low hardness of ~240 Hv. From the hardness contour map, the WM appears to possess a slightly higher surface hardness compared to the BM. The detailed hardness profiles shown in Figure 5b further confirmed this difference. Within approximately 300 μm from the treated surface, the hardness of the WM remains consistently higher than that of the BM. The top surface hardness in the WM exceeds that in the BM by approximately 10%. This result agrees well with the microstructural observations. After tensile deformation to failure, both the WM and BM exhibit a substantial hardness increase, and the hardness gradually approaches the level of the surface layer. The most pronounced hardening occurs in the center region, where the hardness increases significantly from the initial ~240 Hv to ~450 Hv. Such deformation-induced hardening behavior is consistent with previous observations in gradient-structured Cu alloys and steels [33,37,38]. Notably, the WM still shows a slightly stronger hardening response than the BM after tensile deformation, with an average hardness increase of ~25 Hv.

**Figure 6:**
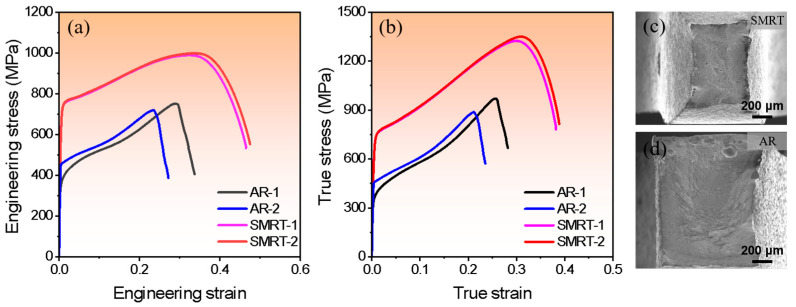
(**a**) Engineering stress–strain curves and (**b**) true stress–strain curves of the SMRT-treated and as-received (AR) samples. SEM images of the fractured surfaces for the (**c**) SMRT-treated sample and (**d**) AR sample after tensile deformation.

The uniaxial tensile results demonstrate that SMRT enhances both the strength and ductility of the welded 304 stainless steel joints. The as-received (AR) welded samples exhibit relatively poor tensile consistency due to the presence of the welded joint, with a yield strength of ~350 MPa and a tensile elongation of ~30% (Figure 6a). After SMRT, the yield strength increases substantially to ~700 MPa, while the ultimate tensile strength reaches ~1000 MPa, accompanied by a simultaneous improvement in ductility. The corresponding true stress–strain curves in Figure 6b reveal that the maximum true stress of the SMRT sample reaches approximately 1350 MPa, which is nearly comparable to that of SMRT-treated non-welded 304 stainless steel reported previously [23]. As shown in Figure 6c,d, the fracture location changes after SMRT. The AR sample fractures directly within the WM without obvious necking, indicating severe strain localization in the WM. In contrast, the SMRT sample fractures in the BM and exhibits pronounced necking before failure. The SMRT-treated welded joint exhibits a noticeable improvement in uniform elongation, indicating delayed strain localization and necking during tensile deformation. Such enhanced deformation stability is particularly advantageous for sheet metal forming operations, especially in the manufacture of laser-welded blanks, where premature necking in the WM often limits formability. The improved uniform elongation demonstrates that the gradient nanostructure not only strengthens the welded joint but also effectively maintains deformation compatibility during plastic deformation. Moreover, the transfer of the fracture location from the WM to the BM, together with the pronounced necking observed before fracture, further confirms that the gradient nanostructure effectively suppresses strain localization in the WM and enhances the overall deformation capability of the welded joint.

### 3.4. Microstructural Evolution During Tensile Deformation

**Figure 7:**
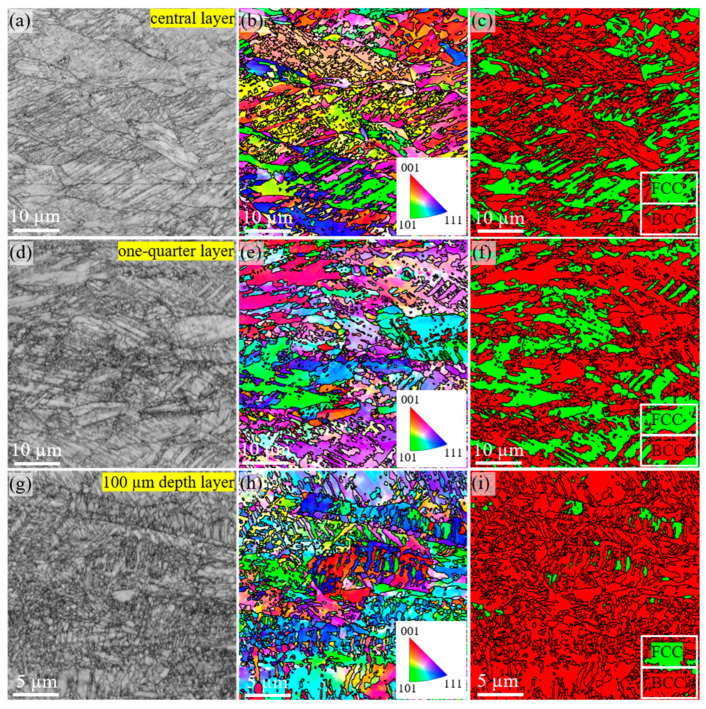
Typical cross-sectional band contrast (BC) images, IPF maps, and phase distribution maps of the WM after tensile deformation (~35% strain): (**a**–**c**) ~100 μm from the top surface, (**d**–**f**) one-quarter thickness layer, and (**g**–**i**) one-half thickness layer.

**Figure 8:**
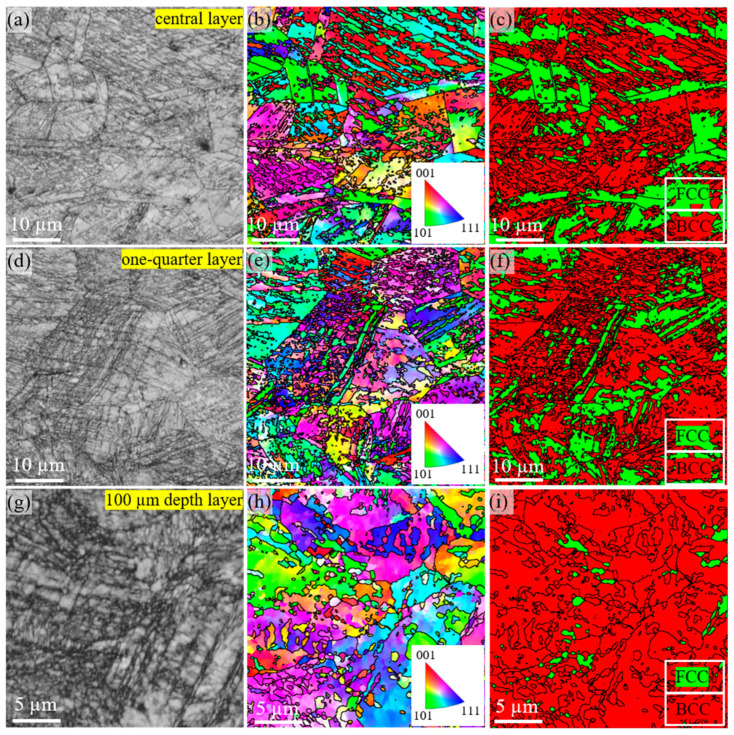
Typical cross-sectional BC images, IPF maps, and phase distribution maps of the BM after tensile deformation (~35% strain): (**a**–**c**) ~100 μm from the top surface, (**d**–**f**) one-quarter thickness layer, and (**g**–**i**) one-half thickness layer.

EBSD characterization further reveals the microstructural evolution and deformation mechanisms in the WM and BM during tensile deformation. As shown in Figure 7, the WM undergoes significant microstructural refinement after tensile deformation, particularly in the central layer and one-quarter thickness region. The deformed microstructure mainly consists of dense ε-martensite laths accompanied by a pronounced increase in the α′-martensite phase fraction. Quantitative phase analysis indicates that the α′-martensite fraction in the central layer increases substantially from the initial ~21% to ~70% after tensile deformation, while the one-quarter thickness region exhibits a similar increase from ~15.5% to ~65%. The comparable martensite fractions in these two regions agree well with the hardness evolution shown in Figure 5b, where both regions exhibit nearly identical hardness after tensile deformation. Notably, the near-surface region of the SMRT-treated welded sample, particularly within approximately 100 μm from the surface, still retains the ability to accommodate plastic deformation through dynamic grain refinement. This result suggests that the gradient nanostructure maintains considerable deformation capability during tensile loading. The BM exhibits a deformation mechanism similar to that of the WM, as shown in Figure 8. Dynamic grain refinement and strain-induced martensitic transformation also dominate the tensile deformation process in both the central layer and one-quarter thickness region. The α′-martensite fraction correspondingly increases to ~72.8% and ~67.5%, respectively. These results indicate that deformation-induced martensitic transformation plays a critical role in strain hardening and deformation accommodation in both WM and BM during tensile deformation.

**Figure 9:**
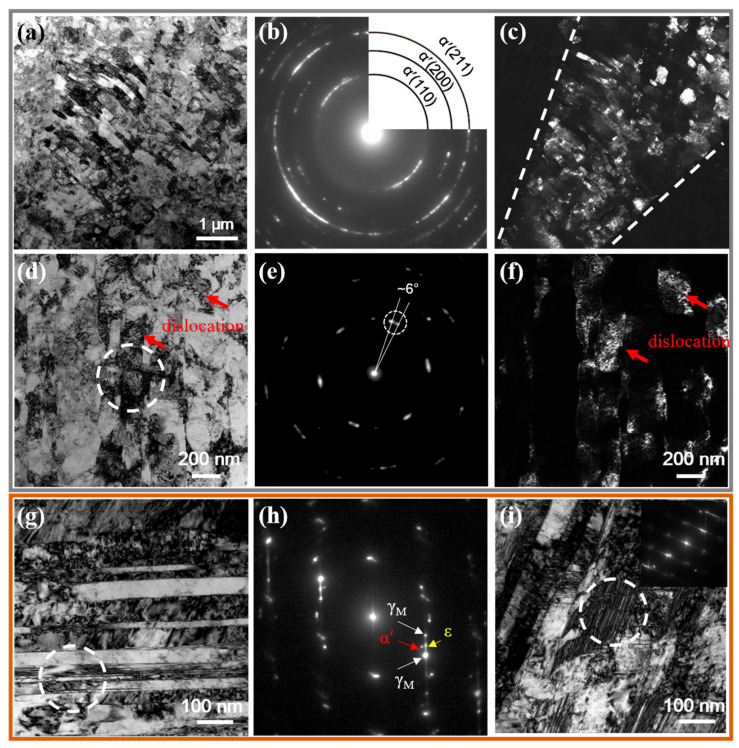
TEM images revealing the microstructural evolution in the WM of the SMRT-treated sample after tensile deformation. (**a**) A bright-field TEM image obtained from the near-surface region at a depth of ~100 μm and (**b**) the corresponding SAED pattern. (**c**) A dark-field TEM image corresponding to (**a**). (**d**) A bright-field TEM image showing low-angle grain boundaries in the near-surface region (~100 μm in depth), and (**e**) the corresponding SAED pattern, indicating a low-angle grain boundary with a misorientation of ~6°. (**f**) A corresponding dark-field image of (**d**). (**g**) A bright-field TEM image obtained from the subsurface region at a depth of ~400 μm and (**h**) the SAED pattern. (**i**) A bright-field TEM image showing high-density stacking faults inside the retained γ-austenite. The white dashed lines in (**c**) indicate the regions exhibiting the preferred orientation. The white circles in (**d**,**g**,**i**) mark the locations where the SAED patterns were obtained.

Figure 9 reveals the deformation mechanisms at the nanoscale through TEM characterization. Due to the thermal input during laser welding, the welded region initially contains coarse austenitic grains. Since the martensitic transformation follows the Kurdjumov–Sachs (K–S) orientation relationship, the martensite nanograins formed during SMRT and subsequent tensile deformation exhibit a strong crystallographic orientation correlation, as shown in Figure 9a–c. Detailed analysis of adjacent martensitic nanograins indicates that the misorientation between neighboring martensite grains remains relatively low, approximately ~6°, corresponding to typical low-angle grain boundaries (Figure 9d–f). Meanwhile, dislocations can still be observed inside the martensitic nanograins, as indicated by the red arrows in Figure 9f, suggesting that plastic deformation is continuously accommodated through dislocation activities within the refined martensitic structure.

At a depth of approximately 400 μm from the treated surface, the refined deformation structures mainly consist of nanoscale ε-martensite lamellae together with progressively formed α′-martensite, as shown in Figure 9h–i. The orientation relationship among γ-austenite, ε-martensite, and α′-martensite satisfies: (111)_γ_//(0002)_ε_//(110)_α′_ and [110]_γ_//[11$\bar{2}$0]_ε_//[111]_α′_, confirming that both ε-martensite and α′-martensite originate from γ-austenite through strain-induced martensitic transformation. In addition, the retained austenitic regions without martensitic transformation still contain a high density of stacking faults. These planar defects further contribute to microstructural subdivision and strengthening during deformation.

### 3.5. Strengthening and Deformation Mechanism

Unlike conventional strengthening strategies that mainly regard the weld metal as the mechanically weakest region [5,7,9,17], the present study demonstrates that laser-welded 304 stainless steel can be effectively strengthened through subsequent gradient surface nanostructuring by SMRT. The superior mechanical performance of the SMRT-treated welded joint primarily originates from the formation of a gradient nanostructure. During SMRT, severe plastic deformation introduces a pronounced strain gradient beneath the treated surface, leading to progressive grain refinement through deformation-induced defects and martensitic transformation. Consequently, a hard nanostructured surface layer and a relatively soft interior are established, producing a typical gradient architecture. Such a heterogeneous structure effectively combines high strength with good tensile ductility through strain partitioning between the surface and the core.

The present results further show that, under identical SMRT conditions, the welded region exhibits a higher fraction of deformation-induced martensite and a finer gradient microstructure than the non-welded region. This difference is likely associated with the microstructural and compositional characteristics introduced by laser welding. In particular, EPMA reveals local fluctuations in the Ni concentration within the weld metal, while STEM-EDS further identifies nanoscale chemical heterogeneity in the form of Cr–Si–O-rich particles. These observations suggest that laser welding generates chemical heterogeneity over multiple length scales. Although the present study does not directly quantify the influence of local Ni fluctuation on austenite stability, previous studies have demonstrated that Ni is the principal austenite stabilizer in metastable 304 stainless steel [39,40,41]. Therefore, the local variation in Ni concentration is likely to modify the stability of γ-austenite, making certain regions more susceptible to deformation-induced martensitic transformation during SMRT.

More importantly, the enhanced martensitic transformation also significantly improves the strain hardening capability during tensile deformation. After SMRT, the welded region no longer acts as the mechanically weakest zone. Instead, the stronger gradient nanostructure and higher martensite fraction effectively suppress strain localization within the WM. During tensile deformation, the retained metastable austenite continuously transforms into martensite, providing sustained strain hardening and plastic strain accommodation. Meanwhile, the gradient structure enables progressive deformation from the hard surface layer to the softer interior, thereby reducing stress concentration and delaying necking [23,42]. The present results provide a new understanding of how the initial microstructure of welded joints affects subsequent gradient surface engineering.

## 4. Conclusions

In this work, SMRT was applied to laser-welded 304 stainless steel joints to improve their mechanical performance through gradient surface nanostructuring. The main conclusions are summarized as follows:

1. Laser welding produced coarse columnar austenitic grains in the weld metal together with microscale Ni compositional fluctuations and nanoscale Cr–Si–O-rich particles, resulting in a chemically and microstructurally heterogeneous weld region.
2. Under identical SMRT conditions, the WM exhibited a stronger tendency for deformation-induced martensitic transformation than the BM, leading to a finer gradient microstructure and a higher near-surface hardness.
3. SMRT remarkably improved the mechanical properties of the welded joints. The yield strength increased from ~350 MPa to ~700 MPa, while an ultimate tensile strength of ~1000 MPa and an elongation of ~40% were simultaneously achieved.
4. During tensile deformation, continuous martensitic transformation and dynamic microstructural refinement provided sustained strain hardening and accommodated plastic deformation. The gradient nanostructure effectively suppressed strain localization and delayed necking, thereby improving the deformation stability of the welded joints.
5. After SMRT, fracture no longer occurred in the WM but shifted to the BM, indicating that gradient surface nanostructuring effectively eliminated the mechanical weakness of the welded region and significantly improved the structural reliability of the welded joint.

Overall, the present work demonstrates that SMRT is an effective approach for enhancing the mechanical performance of laser-welded 304 stainless steel through gradient surface nanostructuring. The initial microstructural characteristics introduced by laser welding appear to further influence the gradient refinement behavior during SMRT, providing new insights into the integration of welding and surface engineering for high-performance welded stainless steel structures.

## Data Availability

The original contributions presented in this study are included in the article. Further inquiries can be directed to the corresponding author.
